# Neoadjuvant Pazopanib Treatment in High-Risk Soft Tissue Sarcoma: A Quantitative Dynamic ^18^F-FDG PET/CT Study of the German Interdisciplinary Sarcoma Group

**DOI:** 10.3390/cancers11060790

**Published:** 2019-06-08

**Authors:** Christos Sachpekidis, Ioannis Karampinis, Jens Jakob, Bernd Kasper, Kai Nowak, Lothar Pilz, Ulrike Attenberger, Timo Gaiser, Hans-Günter Derigs, Matthias Schwarzbach, Peter Hohenberger, Antonia Dimitrakopoulou-Strauss, Ulrich Ronellenfitsch

**Affiliations:** 1 Clinical Cooperation Unit Nuclear Medicine, German Cancer Research Center, 69120 Heidelberg, Germany; ads@ads-net.de; 2 Division of Surgical Oncology and Thoracic Surgery, University Medical Center Mannheim, 68167 Mannheim, Germany; Ioannis.Karampinis@umm.de (I.K.); jens.jakob@med.uni-goettingen.de (J.J.); nowakk@me.com (K.N.); peter.hohenberger@umm.de (P.H.); Ulrich.Ronellenfitsch@uk-halle.de (U.R.); 3 Department of General, Visceral and Child Surgery, University Medical Center Göttingen, 37075 Göttingen, Germany; 4 Interdisciplinary Tumor Center Mannheim, Sarcoma Unit, Mannheim University Medical Center, 68167 Mannheim, Germany; bernd.kasper@umm.de; 5 Department of Abdominal, Vascular and Thoracic Surgery, Romed Klinikum, 83022 Rosenheim, Germany; 6 Medical Faculty Mannheim, University of Heidelberg, 68167 Mannheim, Germany; Lothar.Pilz@medma.uni-heidelberg.de; 7 Institute of Clinical Radiology and Nuclear Medicine, University Medical Center Mannheim, 68167 Mannheim, Germany; Ulrike.Attenberger@medma.uni-heidelberg.de; 8 Institute of Pathology, University Medical Center Mannheim, 68167 Mannheim, Germany; Timo.Gaiser@umm.de; 9 Department of Hematology and Oncology, Klinikum Frankfurt-Hoechst, 65929 Frankfurt am Main, Germany; derigs@klinikumfrankfurt.de; 10 Department of Surgery, Klinikum Frankfurt-Hoechst, 65929 Frankfurt am Main, Germany; Matthias.Schwarzbach@KlinikumFrankfurt.de; 11 Department of Abdominal, Vascular, and Endocrine Surgery, University Hospital Halle, 06120 Halle (Saale), Germany

**Keywords:** soft tissue sarcoma (STS), pazopanib, dynamic ^18^F-FDG PET/CT, SUV, two-tissue compartment model

## Abstract

The outcome of high-risk soft tissue sarcoma (STS) is poor with radical surgery being the only potentially curative modality. Pazopanib is a multikinase inhibitor approved for the treatment of metastatic STS. Herein, in terms of the German Interdisciplinary Sarcoma Group (GISG-04/NOPASS) trial, we evaluate the potential role of kinetic analysis of fludeoxyglucose F-18 (^18^F-FDG) data derived from the application of dynamic positron emission tomography/computed tomography (PET/CT) in response assessment to pazopanib of STS patients scheduled for surgical resection. Sixteen STS patients treated with pazopanib as neoadjuvant therapy before surgery were enrolled in the analysis. All patients underwent dynamic PET/CT prior to and after pazopanib treatment. Data analysis consisted of visual (qualitative) analysis of the PET/CT scans, semi-quantitative evaluation based on standardized uptake value (SUV) calculations, and quantitative analysis of the dynamic ^18^F-FDG PET data, based on two-tissue compartment modeling. Resection specimens were histopathologically assessed and the percentage of regression grade was recorded in 14/16 patients. Time to tumor relapse/progression was also calculated. In the follow-up, 12/16 patients (75%) were alive without relapse, while four patients (25%) relapsed, among them one patient died. Median histopathological regression was 20% (mean 26%, range 5–70%). The studied population was dichotomized using a histopathological regression grade of 20% as cut-off. Based on this threshold, 10/14 patients (71%) showed partial remission (PR), while stable disease (SD) was seen in the rest 4 evaluable patients (29%). Semi-quantitative evaluation showed no statistically significant change in the widely used PET parameters, SUV_average_ and SUV_max_. On the other hand, ^18^F-FDG kinetic analysis revealed a significant decrease in the perfusion-related parameter K_1_, which reflects the carrier-mediated transport of ^18^F-FDG from plasma to tumor. This decrease can be considered as a marker in response to pazopanib in STS and could be due to the anti-angiogenic effect of the therapeutic agent.

## 1. Introduction

The outcome of high-risk soft tissue sarcoma (STS) is poor. Radical surgery, usually in combination with radiotherapy, is the mainstay of treatment and the only potentially curative modality [1]. Surgical removal can, however, be cumbersome due to the large tumor size with infiltration of adjacent structures, and extensive tumor vasculature [2]. In this context, the development and application of a fast acting, preoperative STS therapy that would facilitate tumor resection and at the same time have low toxicity would be of high significance.

Pazopanib is a multikinase inhibitor, approved for the treatment of metastatic STS based on a phase III trial in patients with non-adipocytic STS, who had progressed on at least one prior chemotherapy regimen [3]. Given this proof of efficacy, and its favorable safety profile [4], the German Interdisciplinary Sarcoma Group has very recently published the first results of a single arm phase II trial of preoperative pazopanib therapy in 21 patients with high-risk STS (GISG-04/NOPASS) [5]. Using the metabolic response rate—defined as ≥50% reduction of mean standardized uptake value (SUV_mean_) in post- vs. pretreatment fludeoxyglucose F-18 positron emission tomography/computed tomography (^18^F-FDG PET/CT)—as the primary endpoint, this window-of-opportunity trial showed that preoperative pazopanib is not effective for unselected high-risk STS patients. Nevertheless, metabolic response was observed in a single patient.

In the GISG-04/NOPASS trial, metabolic response was based on estimations of SUV, a semi-quantitative parameter whose calculation requires only static imaging when the tracer ^18^F-FDG has reached equilibrium. SUV represents the tissue activity within a region of interest (ROI) corrected for injected activity and body weight and is the most widely used PET parameter. Nevertheless, the generally accepted method for accurate analysis of ^18^F-FDG metabolism and kinetics is a two-tissue compartment model [6]. A prerequisite for this is the performance of full dynamic PET studies for at least 60 min. In patients with STS, dynamic ^18^F-FDG PET/CT has been prospectively validated as a strong predictor of histopathological response and progression-free survival (PFS), for both neoadjuvant and palliative chemotherapy [7,8,9].

The present analysis is part of the GISG-04/NOPASS trial evaluating the role of neoadjuvant pazopanib in high-risk STS. Herein, we assess the potential role of kinetic analysis of ^18^F-FDG data derived from the application of dynamic PET/CT in response assessment to pazopanib in STS patients scheduled for surgical resection.

## 2. Materials and Methods

### 2.1. Patients-Treatment

Out of the 21 patients enrolled in the GISG-04/NOPASS trial [5], sixteen STS patients (eight female; median age 67.8 years, range 46.3–88.5 years) had evaluable dynamic PET/CT both pre- and post treatment, and were thus enrolled in the analysis. Patients with metastases at presentation were excluded from the study. Treatment consisted of pazopanib 800 mg daily for 21 days as a neoadjuvant therapy before surgery. An interval of 7–14 days between pazopanib therapy completion and surgery was allowed in order to minimize potential perioperative complications due to pazopanib. The herein presented patient cohort has already been studied, and patient data derived from a different analysis have been published elsewhere [5]. The histopathological classification as well as the localization of the sarcomas is presented in Table 1. 

### 2.2. PET/CT

#### 2.2.1. Data Acquisition

All patients underwent PET/CT 14 days prior to and 1–7 days (median 6 days) after pazopanib treatment. A dedicated PET/CT system (Biograph mCT, S128, Siemens Co., Erlangen, Germany) with TruePoint and TrueV, operated in a three-dimensional mode was used. Data acquisition consisted of the dynamic PET/CT (dPET/CT studies) and the static part (whole body PET/CT). dPET/CT studies were performed over the STS area after intravenous administration of ^18^F-FDG for 60 min using a multistep dynamic acquisition over two-bed positions with a total field of view of (21.6 × 2) 43.2 cm. The data were acquired in list mode. A 24-frame protocol (10 frames of 30 s, 5 frames of 60 s, 5 frames of 120 s, and 4 frames of 600 s) was applied. After the end of the dynamic acquisition, whole body, static imaging was performed in all patients with image duration of 2 min per bed position. A low-dose attenuation CT (120 kV, 30 mA) was used for attenuation correction of the dynamic emission PET data and for image fusion. All PET images were attenuation corrected and an image matrix of 400 × 400 pixels was used for iterative image reconstruction. Iterative images reconstruction was based on the ordered subset expectation maximization (OSEM) algorithm with two iterations and 21 subsets as well as time of flight (TOF). 

#### 2.2.2. Data Analysis

Data analysis consisted of visual (qualitative) analysis of the PET/CT scans, semi-quantitative evaluation based on SUV calculations, and quantitative analysis of the dynamic ^18^F-FDG PET data. The assessment of PET/CT scans was performed by two nuclear medicine physicians (Christos Sachpekidis, Antonia Dimitrakopoulou-Strauss).

Qualitative analysis was based on the identification of the STS lesions as sites of increased ^18^F-FDG uptake greater than the background or liver activity. 

Semi-quantitative evaluation was based on volumes of interest (VOIs) and on subsequent calculation of SUV_average_ and SUV_max_. VOIs were drawn with an isocontour mode (pseudo-snake) and were placed over STS lesions [10]. SUV measurements were performed at the 60-min post injection frames.

Quantitative evaluation of the dynamic ^18^F-FDG PET/CT data of the STS lesions was performed using a dedicated software and based on a two-tissue compartment model with a blood component (V_B_), with methods already reported by our group [11,12]. One problem in patients is the accurate measurement of input function, which theoretically requires arterial blood sampling. It has been shown however, that input function can be accurately retrieved from image data [13]. For the input function, the mean value of the VOI data from a large arterial vessel (e.g., aorta or common iliac artery) was used. A vessel VOI consisted of at least seven ROIs in sequential PET images. The recovery coefficient was 0.85 for a diameter of 8 mm. Partial volume correction was performed for small vessels with diameter <8 mm, based on the phantom measurements of the recovery function using dedicated software [14]. 

The application of a two-tissue compartment model leads to the extraction of the kinetic parameters K_1_, k_2_, k_3_, and k_4_ as well as the influx (K_i_) that describes specific molecular processes: K_1_ reflects the carrier-mediated transport of ^18^F-FDG from plasma to tissue, while k_2_ reflects its transport back from the tissue to plasma, and k_3_ represents the phosphorylation rate, while k_4_ the dephosphorylation rate of the glucose analog. The model parameters were accepted when K_1_, k_2_, k_3_, and k_4_ were less than 1 and V_B_ exceeded zero. The unit for the rate constants K_1_, k_2_, k_3_, and k_4_ is 1/min, while V_B_ reflects the fraction of blood within the VOI. Tracer influx (K_i_) is derived from the equation = (K_1_ × k_3_)/(k_2_ + k_3_). The two-tissue compartment model we applied is a modification of the one proposed by Sokoloff et al., which did not take into account the parameters k_4_ and V_B_ [15]. This lack of k_4_ and V_B_ however, leads to different values of the parameters k_1_ and k_3_, since k_1_ is dependent on V_B_ and k_3_ on k_4_. 

Apart from performing compartment analysis, a non-compartment model based on the fractal dimension (FD) for the time activity data was applied. FD is a parameter of heterogeneity of tracer kinetics based on the box counting procedure of chaos theory and was calculated in each individual voxel of a VOI. The values of FD vary from 0 to 2 showing the more deterministic or chaotic distribution of the tracer activity via time, respectively [16].

In addition to the previous analysis, parametric images of the slope and the intercept were calculated based on the dynamic PET (dPET) data by fitting a linear regression function to the time activity data and for each pixel using the PMOD software (PMOD Technologies, Zurich, Switzerland). Parametric imaging is a method for feature extraction, enabling the visualization of single parameters of tracer kinetics, with images of the slope reflecting primarily the trapping of ^18^F-FDG and images of the intercept reflecting the distribution volume of the tracer. These images may be used for the delineation of the malignant lesions and the VOIs placement due to potentially high contrast in the surrounding tissue. Details of this method have been described elsewhere [17,18]. Nevertheless, parametric imaging analysis was not the focus of the present work.

### 2.3. Histological Response

Resection specimens were histopathologically assessed. The percentage of regression grade was recorded as well as data regarding tumor size, resection status (free margins, smallest distance between margin and vital tumor, microscopic and macroscopic infiltration), histological subtype, grade (G1–3), and most prevalent type of regression (hyalinous necrosis, apoptosis, scar tissue, hemorrhagic necrosis) [5] were also recorded. In the present analysis, potential associations between the percentage of regression grade and PET parameters’ changes in response to treatment were assessed.

### 2.4. Statistical Analysis

For continuous variables the mean, standard deviation, 95% confidence interval for the mean, skewness and excess, p-value of the d’Agostino–Pearson test (test for normal distribution), median, range, 95% confidence interval for the median, quartile difference, and lower and upper quartile were given. For dichotomous and ordinal variables, the absolute and relative frequencies, as well as the 95% confidence interval for proportions (Wald method) was used. To compare the values of the PET variables before/after pazopanib treatment the Wilcoxon rank sum test (paired) was performed with the exact p-value, the median difference, and the 95% confidence interval. For the survival analysis, Kaplan–Meier plots were used. Since there were only a few events (deaths or recurrences/relapses), standard statistics was also applied to describe survival. Time to progression (TTP) was measured from first date of PET imaging (entry into the study) to the date of event of local and/or distant relapse. Non-events were censored with the last recorded follow-up date. The same procedure was used for overall survival with the event of death. Univariate comparisons between response groups were performed with unpaired t-tests and rank-sum tests (inclusive the estimated mean/median differences and the 95% confidence intervals of these differences, respectively). 

The level of statistical significance was set to α = 0.05. The statistical tool used was SAS 9.4 (SAS Institute Inc., Cary, North Carolina USA). Graphical data were used in Microsoft Excel. 

## 3. Results

### 3.1. Follow-up Status

Median overall survival (OS) was 3.14 years with a 95% confidence interval of 1.71–3.43 years. In the follow-up, 12/16 patients (75%) were alive without a relapse. Four patients (25%) relapsed, and one patient among them died (Table 2). 

Regarding the four patients with relapse, three patients showed local relapse and distant metastases, while one of them had only distant metastases. Mean time to progression (TTP) after pazopanib treatment was 1.23 years (±0.60 years) and median TTP was 1.46 years with a range 0.31 to 1.70 years.

### 3.2. Histopathological Regression

**Figure 1:**
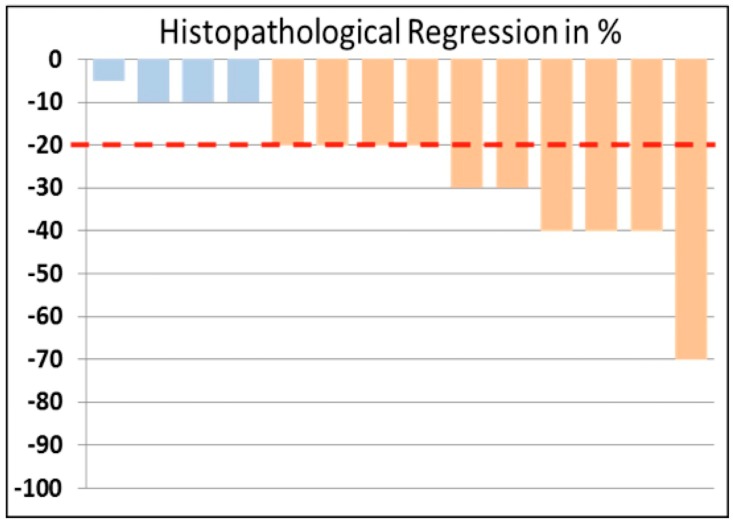
Waterfall plot of the grade of histopathological regression, available for 14 patients. Ten patients had a reduction in vital tumor tissue of >20%, while four patients demonstrated a reduction of less than 20%.

In total, 14 patients were evaluable after pazopanib treatment in terms of histopathology. Histopathological regression was in the mean 26% ± 17% with a median of 20% and a range of 5–70%. The studied population was dichotomized according to the percentage of histopathological regression after pazopanib. Using the 20% regression as threshold, 10/14 patients (71%) showed partial remission (PR; responders), while stable disease (SD; non-responders) was seen in the rest of the 4 evaluable patients (21%). These results are shown in a waterfall plot (Figure 1).

### 3.3. PET/CT Analysis

The results of dPET/CT evaluations before and after pazopanib treatment are presented in Table 3. No statistically significant change of the semi-quantitative parameters SUV_average_ and SUV_max_ was demonstrated. Regarding ^18^F-FDG kinetic analysis, K_1_ was the only parameter that significantly decreased after therapy. All other kinetic parameters did not show any significant change in response to pazopanib. 

**Figure 2:**
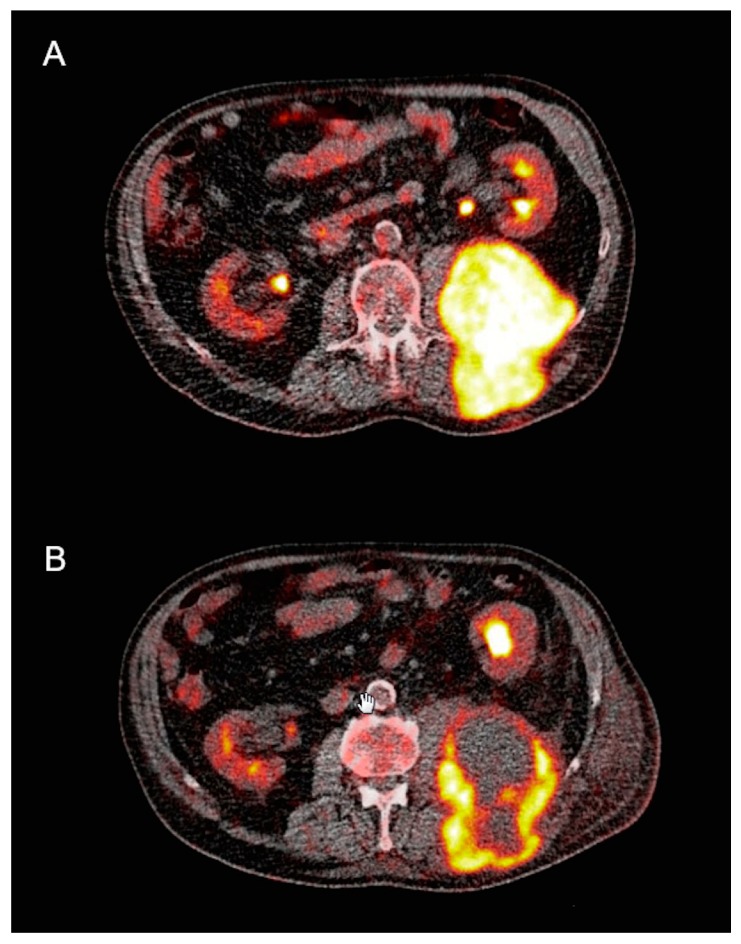
Transaxial fludeoxyglucose F-18 positron emission tomography/computed tomography (^18^F-FDG PET/CT) of an 80-year old male patient with retroperitoneal sarcoma infiltrating the back muscles before (**A**) and after pazopanib therapy (**B**). Clear metabolic remission of the initially intense metabolic lesion with areas of central necrosis in response to pazopanib.

**Figure 3:**
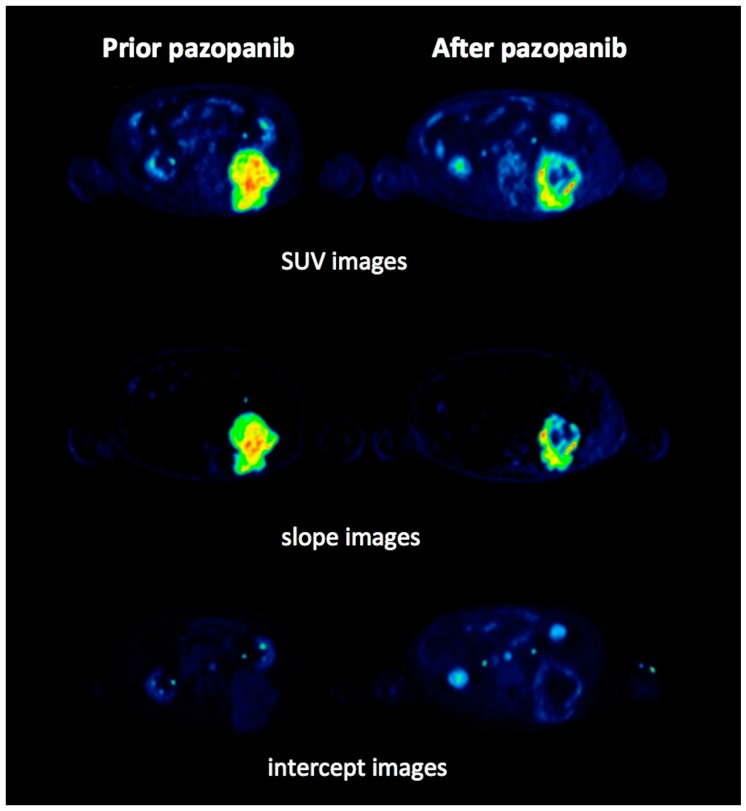
Transaxial fludeoxyglucose F-18 positron emission tomography/computed tomography (^18^F-FDG PET/CT) of the same patient as in Figure 2 before (left) and after pazopanib therapy (right). Standardized uptake value (SUV) images acquired after 60 min of dynamic PET acquisition show a clear metabolic remission of the intense metabolic lesion with central necrosis in response to pazopanib (upper row). Slope parametric images also show initially intense uptake in the area of the tumor, which responds with an essential decrease after therapy due to a decrease in the phosphorylation (middle row). Intercept parametric images demonstrate the tumor very faintly due to the low distribution volume (lower row).

**Figure 4:**
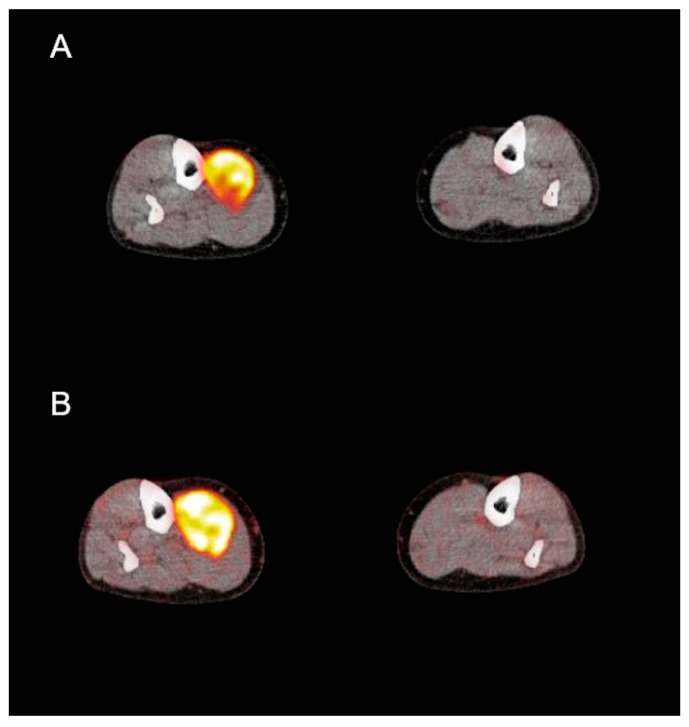
Transaxial fludeoxyglucose F-18 positron emission tomography/computed tomography (^18^F-FDG PET/CT) of a 70-year old female patient with sarcoma of the leg before (**A**) and after pazopanib therapy (**B**). Persistent metabolic activity in the tumor after pazopanib treatment.

Figure 2 and Figure 3 demonstrate an example of a metabolic responder patient after application of conventional, static PET/CT (Figure 2) as well after dynamic PET acquisition involving SUV and parametric images (Figure 3). Figure 4 depicts a metabolic non-responder to pazopanib.

**Figure 5:**
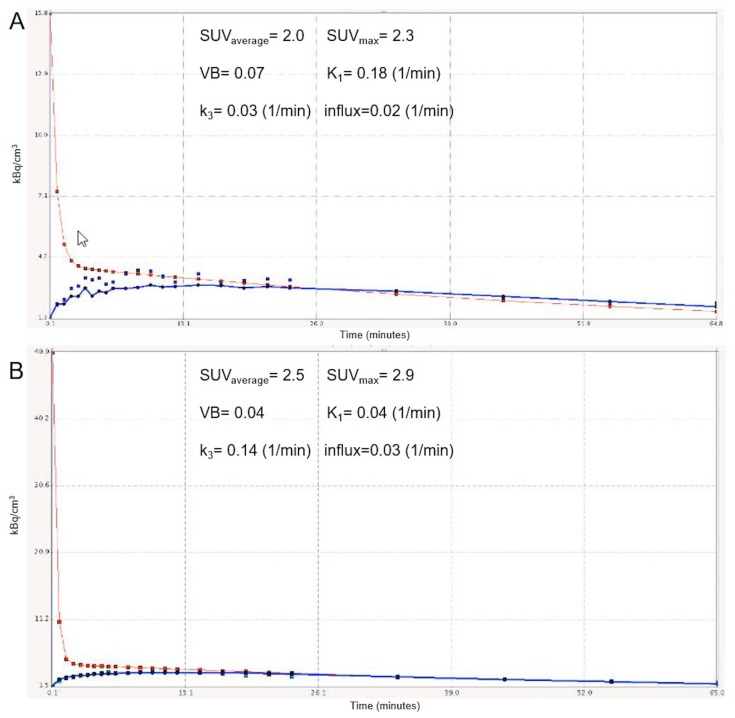
Time-activity curves (TACs) derived from dynamic positron emission tomography/computed tomography (PET/CT) studies of a retroperitoneal soft tissue sarcoma (STS) before (**A**) and after (**B**) pazopanib therapy (y-axis: kBq/cm3; x-axis: minutes). The TACs are derived from volumes of interest (VOIs) corresponding to the tumor (blue curve) and the descending aorta (red curve). The tumor curves show an increase in the fludeoxyglucose F-18 (^18^F-FDG) accumulation in the tumor VOI during the 60 min of dynamic PET acquisition (reflected by an increase in standardized uptake value-SUV values), but at the same time a decrease in the carrier-mediated transport of the tracer from plasma to the tumor (reflected by a decrease in K_1_) in response to pazopanib. VB: blood volume.

Figure 5 depicts the time-activity curve (TAC) of ^18^F-FDG in a STS before and after treatment.

We further performed comparisons of the PET parameters between the groups of responders (PR) and non-responders (SD), according to the histopathological criteria applied in the study. Unpaired test procedures (t-test/rank-sum Wilcoxon test as appropriate) showed no statistically significant differences between the two groups in response to the treatment. No significant correlation between histopathological regression and ^18^F-FDG kinetics response was observed. 

Finally, TTP data were also studied in association with ^18^F-FDG PET/CT data. Similarly to histopathological regression, no association between tracer kinetics and time to tumor relapse was observed.

## 4. Discussion

The current study was designed to analyze the potential role of both the semi-quantitative (SUV) and quantitative/kinetic evaluation of ^18^F-FDG PET for response assessment of neoadjuvant pazopanib treatment in STS patients. This analysis took place in the framework of a phase II window-of-opportunity trial of the German Interdisciplinary Sarcoma Group (GISG-04/NOPASS). 

No statistically significant changes of the semi-quantitative parameters SUV_average_ and SUV_max_ in response to pazopanib could be demonstrated. This finding is expected, given the recently published results of our group, which revealed a mean decrease of 6% in SUV_average_ of post- vs. pretreatment PET/CT in the same patient cohort [2]. The primary endpoint of that analysis was based on a definition of metabolic response as >50% decrease in SUV_average_, leading to the characterization of only one subject as the metabolic responder. It could be argued that the applied SUV threshold of 50% was rather high, and the application of a lower one would have led to a higher metabolic response rate. Nevertheless, a reduction of the threshold to 40%—as suggested by Schuetze et al. [19]—would have yielded the same metabolic response rate. Respectively, the application of the thresholds suggested by the two most widely used PET criteria (positron emission tomography response criteria in solid tumors-PERCIST and European Organisation for Research and Treatment of Cancer-EORTC) for definition of partial metabolic response would not have led to essentially different results. In particular, the usage of a 30% decrease of SUV–according to the PERCIST criteria [20]—would also classify only one patient as the metabolic responder, while a reduction of the SUV threshold to 25%—according to the EORTC criteria for PET [21]—would have resulted in three metabolic responders and thus still in a formally negative trial result.

SUV is the most widely used method for quantification of PET data, since its calculation requires only static imaging usually 60 min p.i. However, the ^18^F-FDG uptake 60 min after tracer injection is the result of a dynamic process. One important aspect of PET is the possibility of performing accurate, noninvasive quantitative measurements of tracer concentration in patients. The generally accepted method for accurate analysis of ^18^F-FDG metabolism and pharmacokinetics is a two-tissue compartment model [6], which requires, however, the performance of a dynamic PET (dPET) study with duration of 60 min, in addition to the regular static PET/CT scan. This is of course more time consuming, both for the patient and the institution, and thus is mainly limited to research centers, as ours. Particularly in STS, the application of dPET and the subsequent acquisition of ^18^F-FDG kinetic information have been shown useful in early prediction of chemosensitivity in the neoadjuvant context [8]. 

^18^F-FDG kinetic analysis before and after pazopanib treatment revealed a significant decrease of the parameter K_1_, which reflects the carrier-mediated transport of the tracer from plasma to tumor, in response to therapy. Although not in line with the results of semi-quantitative (SUV) analysis, this decrease in K_1_ could be partly explained by the mechanism of action of pazopanib. Pazopanib exerts its action through inhibition of growth factor receptors associated with angiogenesis and tumor cell proliferation [22]. Given that K_1_ is a perfusion-related parameter [23], it could be suggested that the anti-angiogenic effect of pazopanib is responsible for this finding. Moreover, this result is in accordance with that published by Dimitrakopoulou-Strauss et al. in a group of 31 STS patients receiving neoadjuvant chemotherapy consisting of etoposide, ifosfamide, and doxorubicin, who were also followed by dPET; the patients in that study also showed a significant decline in K_1_ after two chemotherapeutic cycles [8]. Based on these results, the parameter K_1_ could be potentially used as an early response marker of the anti-angiogenic effect of pazopanib with changes of this parameter being used for therapeutic management decisions, of course after taking into account other tumor-related parameters. However, long-term follow-up is required to confirm the herein presented results. 

On the other hand, the phosphorylation rate of ^18^F-FDG (k_3_), its influx (K_i_), and the fractal dimension (FD) did not show any significant change. This is in line with the lack of significant tracer uptake (SUV) decrease in response to treatment. Although the reason for this lacking response is unknown, apart from the small sample size, a targeted anti-angiogenic action of pazopanib without a pronounced and fast effect on tumor glucose metabolism could be hypothesized. 

Since there is no uniformly accepted gold standard to define histopathological regression, a threshold of 20%—based on the calculated median histopathological regression grade and in line with the first published GISG-04/NOPASS study [5]—was applied in order to dichotomize the studied population into responders and non-responders after pazopanib. The comparison of the PET parameters between these groups showed no statistically significant differences both at baseline and as response to treatment. Moreover, no significant correlation between histopathological regression and ^18^F-FDG kinetics response was observed, which, however may be due to the small sample size.

Although the follow-up time was rather short, TTP data were also utilized in the present analysis to search for potential associations between ^18^F-FDG kinetics and disease relapse. Four of the studied patients (25%) relapsed with a mean TTP of 1.23 years; three of them showed local relapse and distant metastases, while one of them showed only distant metastases. In line with the previously presented results, no correlation between tracer kinetics and TTP was observed, which could be attributed to the small sample size and the limited number of progression events.

The main limitation of our analysis is the small number of patients evaluated, rendering a more robust statistical evaluation difficult. This is because patient enrolment in the trial was stopped based on the result of a futility analysis presented in the first results published by our group [5]. 

## 5. Conclusions

The results of the present study show that the widely used PET parameter SUV may not be the adequate measure to assess the response to neoadjuvant pazopanib treatment in STS patients. On the other hand, the perfusion-related, kinetic parameter K_1_—reflecting the carrier-mediated transport of ^18^F-FDG from plasma to tumor—significantly decreased during pazopanib treatment. This finding could be due to the anti-angiogenic effect of the agent. Based on these findings, K_1_ could be potentially used as an early response marker of the pazopanib treatment efficacy, with changes of this parameter being used for therapeutic management decisions. However, long-term follow-up is required to confirm the herein presented results.

## Figures and Tables

**Table 1:** Histopathological characteristics and localization of the soft tissue sarcoma (STS) of the 16 studied patients.

**Histology**	**No.**	**%**
Dedifferentiated liposarcoma	8	50%
Undifferentiated pleomorphic sarcoma	2	12.5%
Fibrohistiocytic sarcoma	1	6.25%
Leiomyosarcoma	1	6.25%
Malignant peripheral nerve sheath tumor	1	6.25%
Myxoid liposarcoma	1	6.25%
Pleomorphic liposarcoma	1	6.25%
Synovial sarcoma	1	6.25%
**Localization of the primary**	**No.**	**%**
Retroperitoneal	6	37.5%
Left thigh	4	25%
Right shank	2	12.5%
Left gluteal	1	6.25%
Left inguinal	1	6.25%
Pelvis	1	6.25%
Right middle abdomen	1	6.25%

**Table 2:** Follow up status of the 16 studied patients treated with pazopanib.

Follow Up Status	No.	%
Alive without relapse	12	75%
Alive with relapse	3	18.75%
Dead	1	6.25%

**Table 3:** Descriptive statistics of mean and median values prior and after pazopanib therapy for the ^18^F-FDG semi-quantitative and quantitative parameters in STS. The values of parameters K_1_, k_2_, k_3_, k_4_ and influx are 1/min. SUV values, blood component (V_B_), and fractal dimension (FD) have no units.

Parameter	Mean Prior	Median Prior	Mean After	Median After
SUV_average_	5.7	3.8	5.0	4.1
SUV_max_	10.2	7.3	8.0	6.5
V_B_	0.10	0.06	0.07	0.03
K_1_ * (1/min)	0.26	0.19	0.16	0.12
k_2_ (1/min)	0.35	0.33	0.31	0.25
k_3_ (1/min)	0.11	0.10	0.13	0.14
k_4_ (1/min)	0.03	0.03	0.05	0.02
Influx (1/min)	0.06	0.04	0.04	0.03
FD	1.18	1.16	1.17	1.16

* Significant probabilities (*p* < 0.05). SUV, standardized uptake value; FD, fractal dimension.
